# Occupational disease claims and non-occupational morbidity in a prospective cohort observation of nickel electrolysis workers

**DOI:** 10.1038/s41598-022-11241-5

**Published:** 2022-04-30

**Authors:** Sergei Syurin, Denis Vinnikov

**Affiliations:** 1 Northwest Public Health Research Center, Saint Petersburg, Russian Federation; 2 Al-Farabi Kazakh National University, Al-Farabi Kazakh National University, 71 al-Farabi Avenue, Almaty, 050040 Kazakhstan; 3 Peoples’ Friendship University of Russia (RUDN University), Moscow, Russian Federation

**Keywords:** Risk factors, Epidemiology

## Abstract

Exposure to nickel aerosol in the nickel production is associated with greater occupational risk, yet little is known how many workers will develop an occupational disease and claim compensation. The aim of this analysis was to prospectively observe a cohort of nickel electrolysis workers and quantitatively assess confirmed occupational disease claims. We observed a cohort of nickel electrolysis workers (N = 1397, median age 39, 68% males) from 2008 till 2020 in one of the largest nickel producers in the Russian High North. Cumulative incidence of confirmed occupational disease claims in seven occupational groups, including electrolysis operators, hydrometallurgists, crane operators, final product cleaners, metalworkers, electricians and ‘other’ was analyzed and supplemented with Cox proportional hazards regression, yielding hazard ratios (HR) with their 95% confidence intervals (CI) of occupational disease claims for each group. N patients with occupational disease claims varied from 1 in 2016 to 22 in 2009, and in total 87 patients developed one or more occupational diseases (cumulative incidence 6.2%, p < 0.001 between seven groups). Accounting for 35,527 person-years of observation in total, cleaners exhibited the greatest risk (HR 2.58 (95% CI 1.43–4.64)), also adjusted for smoking, number of non-occupational diseases and group 2 (hydrometallurgists). Smoking was independently associated with having an occupational disease claim in all groups (p < 0.001), as was the number of non-work-related diseases in six groups of seven. Despite consistent improvement in the exposure control measures in nickel production, occupational morbidity persists. More effort is needed to reduce exposure in final product cleaners.

## Introduction

Occupational exposure to nickel is quite prevalent, but not limited to, welders, metalworking and nickel/cobalt mining and production. Levels of exposure may vary dramatically in welders, metalworkers and those employed in nickel mining and nickel smelting^1–5^. Moreover, exposure can also be affected by the production method and the use of technologically advanced extraction and processing approaches lately. Workers in the nickel production may be exposed to a combination of water-soluble nickel and its non-soluble salts in dust; however, occupational exposures in this industry is not limited to nickel, but also embraces noise, physical overload and high-temperature environment^2^.

Health effects of such exposure in these occupational groups have been widely described in the medical literature and is usually grouped to toxic, allergic and carcinogenic effects^6,7^. Non-carcinogenic effects of nickel include nickel toxicity^8^ and allergic effects^9^; however, cancer associated with exposure to nickel may have direct occupational implications^10,11^. Respiratory cancer risk is elevated in electrolysis workers involved in the electrolytic extraction of pure copper and nickel and exposed to water-soluble salts of sulfuric and hydrochloric acids^12^; in those working in furnace departments; and in workers producing water-soluble nickel and copper salts (sulphates) from aqueous solutions^13^. The findings of further reports in larger cohorts confirmed these associations and expanded the list of nickel-associated cancers to sinonasal cancer^14^, kidney cancer^15^, and rectum^16^.

Health effects of exposure to nickel in nickel production are not limited to cancer. Common chronic work-related respiratory diseases, such as chronic bronchitis, chronic obstructive pulmonary disease and asthma were also found in these workers^17^. However, other chronic inflammatory diseases are poorly studied in nickel mining and refinery workers. In addition, claiming an occupational disease in a worker in some countries, such as the Russian Federation, where the confirmation of an occupational disease is subject for further compensation, is also indicative of an association of exposure with a disease in the workplace. Occupational disease claims as an alternative approach to depict occupational morbidity in these occupations have never been systematically analyzed and reported. We, therefore, aimed to prospectively observe a cohort of nickel electrolysis workers and quantitatively assess confirmed occupational disease claims.

## Results

We formed a fixed cohort of nickel electrolysis workers, including seven occupational groups, at their annual screening in 2008 (N = 1397, 68% men). The median age at inclusion was 39 (IQR 16) years, significantly different between occupational groups with the youngest workers in groups 1 and 4 and most senior employees in the “other” occupational group (Table 1). Concurrently, we found the similar trend of differences in the work duration. Current daily cigarette smoking prevalence was high at inclusion (51% of the cohort), almost twice more in men compared to women (60% vs. 32%, p < 0.001). Eight pack-years was the median smoking intensity and duration, significantly greater in groups with older worker population, and in men (median 9 (IQR 10)) compared to women (median 6 (IQR 5.9)), p < 0.001. Only 12% of the cohort in 2008 did not have any diagnosis with no difference between occupational groups (Table 1). Subjects not classified as ‘healthy’ had on average two diagnoses.

**Table 1:** The overall descriptive data of the cohort and occupational groups.

	Overall	Group 1	Group 2	Group 3	Group 4	Group 5	Group 6	Group 7
N (%)	1397 (100)	366 (26)	389 (28)	104 (7)	105 (7)	184 (13)	102 (7)	147 (12)
Males, N (%)	959 (68)*	203 (55)	264 (68)	6 (6)	80 (77)	185 (100)	102 (100)	119 (82)
Age, years	39 (16)*	36 (17)	40 (16)	38 (12)	36 (17)	40 (18)	37.5 (14)	43 (12)
Years in occupation	12 (12)*	10 (8)	12 (14)	13 (12)	11 (14)	12 (11)	14 (14)	15 (12)
Current smokers, N (%)	712 (51)*	176 (48)	197(51)	46(44)	57(54)	115(63)	58(57)	63 (43)
Pack-years in smokers	8 (9)*	7 (8)	9 (10)	5 (5)	7.5 (8)	10 (12)	9 (9)	11 (9)
Healthy subjects, N (%)	172 (12)	48 (13)	37 (10)	18 (17)	13 (12)	28 (15)	12 (12)	16 (11)
N diagnoses in each subject, median (IQR)	2 (3)	2 (3)	2 (3)	2 (3)	3 (3)	2 (3)	2 (2)	2 (3)

*p < 0.05 in Kruskall-Wallis test of comparing 7 groups oк from 2*7 χ2 test; group 1—electrolysis operator; group 2—hydrometallurgist; group 3—crane operator; group 4—finished product cleaner; group 5—metalworker; group 6—electrician; group 7—other occupations.

At the annual screening in 2008, the overall number of diagnoses in group 1 was 1035; group 2—1006; group 3—517; group 4—261; group 5—283; group 6—251; and finally, 391 diagnoses in group 7. In the sample of 1397 workers, the total count of diagnoses was 3744. Almost one-fourth of all diagnoses were musculoskeletal diseases (Table 2), followed by ocular diseases, whereas respiratory and cardiovascular diagnoses together at the screening were identified in 25% of subjects. Diseases with less than 2% prevalence did not exhibit significant difference between occupational group because of dramatic fall in statistical power in the χ2 test.

**Table 2:** The number and percent of selected disease classes identified at the annual screening in seven groups of workers.

Disease class	Total (n = 3744)	Group 1 (n = 1006)	Group 2 (n = 1035)	Group 3 (n = 261)	Group 4 (n = 283)	Group 5 (n = 517)	Group 6 (n = 251)	Group 7 (n = 391)
Musculoskeletal*	862(23.0)	281 (27.9)	214 (20.7)	79 (30.3)	56 (19.8)	104 (20.1)	51 (20.3)	77 (19.7)
Ocular*	712(19.0)	180 (17.9)	201 (19.4)	39 (14.9)	55 (19.4)	103 (19.7)	52 (20.7)	82 (21.0)
Respiratory*	474(12.7)	115 (11.4)	123 (11.9)	31 (11.9)	44 (15.5)	80 (15.5)	33 (13.1)	48 (12.2)
Cardiovascular*	372 (9.9)	83 (8.3)	113 (10.9)	16 (6.1)	23 (8.1)	66 (12.8)	28 (11.2)	43 (10.9)
Endocrine and metabolic*	271 (7.2)	72 (7.7)	77 (7.4)	18 (6.9)	25 (8.8)	30 (5.8)	15 (6.0)	34 (8.7)
Infectious and parasitic*	207 (5.5)	34 (3.4)	72 (7.0)	15 (5.7)	21 (7.4)	38 (7.4)	9 (3.6)	18 (4.7)
Gastrointestinal*	232 (6.2)	57 (5.7)	71 (6.9)	12 (4.6)	17 (6.0)	28 (5.4)	22 (8.8)	25 (6.5)
Genitourinary*	217 (5.8)	65 (6.5)	57 (5.5)	27 (10.3)	15 (5.3)	19 (3.7)	12 (4.8)	22 (5.7)
Skin*	172 (4.6)	63 (6.3)	39 (3.8)	8 (3.1)	13 (4.6)	22 (4.3)	12 (4.8)	15 (3.9)
Ear*	75 (2.0)	16 (1.6)	28 (2.7)	2 (0.8)	2 (0.7)	15 (2.9)	5 (2.0)	7 (1.7)
Neoplasms	69 (1.8)	15 (1.5)	24 (2.3)	8 (3.1)	4 (1.4)	5 (1.0)	7 (2.8)	6 (1.5)
Trauma, intoxications and other external	18 (0.5)	4 (0.4)	8 (0.8)	1 (0.4)	1 (0.4)	2 (0.4)	1 (0.4)	1 (0.2)
Nervous system	35 (0.9)	12 (1.2)	3 (0.3)	3 (1.1)	5 (1.8)	3 (0.6)	2 (0.8)	7 (1.9)
Other	28 (0.7)	9 (0.9)	5 (0.5)	2 (0.8)	2 (0.7)	2 (0.4)	2 (0.8)	6 (1.8)

*p < 0.05 from 2*7 χ2 test; group 1—electrolysis operators; group 2—hydrometallurgists; group 3—crane operators; group 4—finished product cleaners; group 5—metalworkers; group 6—electricians; group 7—other occupations.

We then followed the sample for twelve consecutive years with 35,527 person-years in total, of whom N = 150 (10.7%) dropped out of the cohort at any time after 2008 (death, move, retirement). We found that the number of newly identified and verified occupational disease claims ranged from 1 (in 2016) to 44 (in 2009). Consistent with the change in the number of such new cases of occupational disease, the number of patients also varied from the least 1 in 2016 to 22 in 2009, because one patient may have had more than one occupational disease claim. Eighty-seven patients in total claimed and confirmed an occupational disease during 12 years of follow-up (cumulative incidence 6.2%). Twenty-four of these workers were from group 1; 17 from group 2; 6 from group 3; 14 from group 4 and 5 each; 5 from group 6; and 7 from group 7. Thus, the cumulative incidence of occupational claims during 12 years of follow-up for groups was 7%; 4%; 6%; 13%; 8%; 5% and 5%, accordingly (p = 0.04 for 2*7 χ2 test). The cumulative incidence ratio for group 1 was 1.07; 0.73 for group 2; 0.92 for group 3; 2.36 for group 4; 1.46 for group 5; 0.83 for group 6; and 0.74 for group 7. The overall number of diagnoses of occupational disease confirmed in the current cohort was 17. The most prevalent occupational diagnoses was chronic bronchitis (in 57 subjects), followed by bronchial asthma (in 26 subjects) and sensorineural deafness (15 cases), whereas the least prevalent diseases were osteoarthritis (in 2 subjects), contact dermatitis (in two subjects) and malignancy (2 cases).

We then tested selected predictors of having occupational disease claim at any time follow-up in the univariate analyses. In such univariate analyses, sex was not associated with the outcome (69% men in the group with a disease and 66% men in the group with no occupational disease, p = 0.52), but smoking as a binary variable was (64% smokers in the group with a disease and 50% smokers in the alternative group, p < 0.001). Such significant association of smoking with the outcome remained highly significant when smoking was considered as a continuous variable of pack-years. In addition, we found significant association of general non-occupational morbidity, as yes/no with regard to any disease and the number of diagnoses, with getting any occupational disease claim at any time of employment.

When an occupational group was tested in a Cox regression analysis, accounting for the time elapsed from work commencement to the occupational disease claim and adjusted for smoking intensity and number of non-occupational diagnoses at the annual screening (Model 1), we found greater risk for group 4 and protective effect for group 2 (Table 3). In addition, smoking was independently associated with having an occupational disease claim at follow-up in all 7 groups analyses (p < 0.001), whereas the number of non-work-related disease at annual screening was able to predict occupational disease claims in six groups of seven. When we then applied Model 2, in which two occupational groups (group 2 and group 4) were adjusted for all the confounders of Model 1 and additionally adjusted for each other, only group 4 was associated with occupational disease claims (HR 2.58 (95% CI 1.43–4.64)). We found almost a fourfold increase in the risk of outcome when smoking was combined with group 4 (HR 3.83 (95% CI 1.92–7.66), p < 0.001). Finally, we performed similar adjusted analysis for the claim of 17 specific occupational diseases as opposed to any work-related disease as a whole and found that group 7 workers claimed more lumbar radiculopathy (HR 5.53 (95% CI 1.07–28.6)) and chronic rhinopharyngolaryngitis (HR 4.39 (95% CI 1.20–16.98)). Chronic bronchitis was the most prevalent diagnosis claim, but it’s claim was not associated with any group because of similar distribution between these groups.

**Table 3:** Cox proportional hazards analysis for seven occupational groups (Model 1).

Occupational group	HR with 95% CI	Smoking pack-years beta with 95% CI	Number of non-work-related diseases beta with 95% CI
Group 1	NS	0.04 (0.02–0.07)	NS
Group 2	0.55 (0.32–0.94)	0.04 (0.02–0.07)	0.09 (0.01–0.17)
Group 3	NS	0.04 (0.02–0.07)	0.09 (0.01–0.17)
Group 4	2.93 (1.65–5.21)	0.05 (0.02–0.07)	0.09 (0.01–0.18)
Group 5	NS	0.04 (0.02–0.07)	0.09 (0.01–0.18)
Group 6	NS	0.04 (0.02–0.06)	0.09 (0.01–0.18)
Group 7	NS	0.04 (0.02–0.07)	0.09 (0.01–0.17)

Each group test is adjusted for smoking pack-years, number of non-work-related diseases and time elapsed since work start to censor.

*HR* hazard ratio, *CI* confidence interval, *NS* non-significant.

## Discussion

This is the first report of occupational diseases claims in nickel electrolytical refinery using prospective cohort design, which uncovers the full range of diagnoses, found and confirmed as work-related in a large cohort of electrolysis workers. Nickel electrolysis workers in the Russian High North refinery claimed altogether 17 work-related diagnoses, of which chronic bronchitis, asthma and sensorineural deafness were the most prevalent. Within the cohort, final product cleaners had the greatest risk of acquiring an occupational disease, independent of smoking and non-occupational morbidity.

Respiratory diseases as the leads in the occupational burden in our nickel electrolysis workers^18^ are consistent with the overall assumption that aerosol inhalation is the dominating occupational exposure in this industry^19^. Exposure to vapors, gases, dusts and fumes has been demonstrated to explain a significant fraction of respiratory disease in a population since long time ago^20^; however, it is only recently that studies from the Russian Federation emerged in the English literature^21^. Population attributable fraction % (PAF%) found is this meta-analysis and equaled 6%, was indeed an underestimate of the burden of occupational respiratory disease because of the likely exposure classification bias, when exposure may be underestimated or reported inaccurately. In the Russian High North small towns, where most population is employed by the mines or smelters, this PAF% may be much greater.

The elucidated higher risk in the final product cleaners needs further investigation with deeper insight in the exposure assessment of these workplaces. We believe this greater burden of occupational disease in this group was due to a combination of specific exposures. In addition to noise and water-soluble nickel aerosol, cleaners were exposed to nickel dust, which can explain more respiratory diagnoses. Because there may be a dramatic difference in the penetrative potential of particulate matter of various aerodynamic diameter, we call for higher quality studies of inhalable vs. fine particulate matter concentrations in the nickel production. We have not found studies published on the industrial hygiene data from the company with focus on particle diameter and even chemical composition. Our data may be the first presentation of the higher risk in final product cleaners, which should guide further targeted exposure assessment studies in this occupational group.

Cigarette smoking prevalence was high in the current cohort, consistent with other occupational settings with male dominance in mining and production^4,22,23^. Our finding of further increase in risk in smoking workers when added to an occupational hazard necessitates efficient and timely smoking initiation prevention programs, as well as affordable smoking cessation programs in the workplace. Evidence from many countries have confirmed the effect of smoking cessation programs in various occupational groups^24–26^, yet remain infrequently offered to workers and enforced by the companies. Comprehensive smoking reduction and/or cessation programs must be prioritized by the management in production sites, whereas the information on the negative health effects of smoking and ways to quit should be repeatedly conveyed to workers at the annual screening.

Our study has distinct strengths and limitations. Data collected from one of the largest nickel producers, in which productions is localized in one venue, allowed to compare multiple occupational groups. Secondly, observational cohort design with a follow-up to 12 years and accounting for the overall years in service in proportional hazards regression models enabled to speculate over the causality of identified outcomes. Thirdly, in contrast to other reports in nickel electrolysis cohorts, we could with scrutiny accumulate data on the wide range of non-occupational, somatic diseases at the annual screening and consider these in the regression models. However, the current cohort was only built on electrolytical extraction workers and did not include mining stage or even other industries and population, when comparisons could be made with workers with contrasting exposures or no exposures., which is a clear limitation. In addition, the workforce of only one producer could participate, limiting our sample. Furthermore, no reliable and accurate exposure assessment data are available, which hampers risk assessment based on actual exposure rather than on an occupation or a position.

In conclusion, this is the first presentation of the prospective cohort observation of occupational disease claims in nickel electrolysis workers, showing that respiratory disease and sensorineural deafness as the most prevalent confirmed occupational diseases reflect typical exposures in nickel production. Consistent with combined exposure to nickel water-soluble aerosol, nickel dusts and musculoskeletal load present in final product cleaners, workers in this occupation exhibit the higher rates of occupational disease claims. Addressing these hazards in the workplace of cleaners and other occupational groups could reduce occupational morbidity and support occupational longevity in nickel production in the future.

## Materials and methods

### Study protocol and venue

This was a prospective cohort observation of nickel electrolysis workers. The study was approved by the Committee of Bioethics of the Northwest Public Health Research Center and was conducted in accordance with the relevant guidelines and regulations. Informed consent was obtained from all subjects. Moreover, data for this analysis were transferred from the medical data registry fully anonymized. Subjects were enrolled during their annual medical screening for fitness to work in 2008, for which a worker is referred by the human resources department to a designated outpatient governmental medical facility in the place of residence. The scope of such screening along with the list of examinations and tests is informed by the governmental Order of the Ministry of Healthcare of the Russian Federation. Time 0 of our cohort observation, annual screening of 2008 was targeted and expanded to a pulmonologist, endocrinologist, gastroenterologist and urologist as addition to routine clinical tests and eight specialists of a panel. They all should suspect, refer to further examination and confirm clinical diagnoses and decide whether an employee is fit to work with such diagnoses and under documented exposure in the workplace.

Full nickel production cycle includes mining, ore transportation, milling, ore dressing, pyrometallurgical processing, electrolysis and final production. The company in the current presentation is located in the Russian High North. In 2019, the company produced 166265 tons of nickel (approximately 10% of the total world production) with gradual yearly production increase. It is one of the largest nickel producers in the world and is involved in all stages of nickel production, while we analyze occupational disease claims in the electrolysis production stage not comparing them with mining and other processes due to largely contrasting exposures and technologies of the multiple-staged nickel production. Nickel electrolysis in the company is confined to two similar workshops, each consisting of three departments, such as hydrometallurgic, electrolysis and final production. Departments may differ with regard to the levels of exposure and aerosol chemical composition. In addition to aerosol, some occupations exhibit musculoskeletal overload due to weightlifting and awkward working posture.

### Participants

All enrolled subjects were employed for the processes at the stage of metal nickel electrolysis production. Subjects’ demographic data, smoking behavior, detailed work history, including years in occupation and duration of work on a current position as well as clinical data were routinely collected at the annual screening. Informed by specific exposures and production technology, subjects in the current cohort were grouped into seven occupational groups, of which the first six were specific occupations, whereas the seventh group, ‘other’, consisted of workers of various occupation, not categorized otherwise. These groups included electrolysis operators (group 1); hydrometallurgists (group 2); crane operators (group 3); finished product cleaners (group 4); metalworkers (group 5); electricians (group 6); and ‘other occupations’ (group 7).

Electrolysis operators (group 1) work 8-h shifts and are in charge of filling electrolysis baths with a solution, controlling electrolyte circulation velocity, washing baths and nickel sedimentation surfaces. Exposure to nickel aerosol is up to 100% of working time, whereas up to 80% of time workers are in vertical posture. In addition, workers are overexposed to noise. Company’s industrial hygiene data assume overexposure to nickel aerosol in the breathing zone (mean concentration from two workshops 0.148 mg/m^3^ (occupational exposure limit of water-soluble nickel (OEL) 0.005 mg/m^3^), peak concentration equaled 1.383 mg/m3) and cobalt (mean concentration 0.010 mg/m^3^ (OEL 0.05 mg/m^3^)), but exposure to chlorine and sulfuric acid are within OEL.

Hydrometallurgists (group 2) also work 8-h shifts controlling solution filtration in the vacuum filters visually, also engaged in pulp suction from the filters, pulp supply, water and sulfuric acid supply to reactors, visual control over the equipment and all pipeline maintenance. These workers are overexposed to noise, 80% time are in standing position, exhibit greater physical overload and overexposure to nickel (mean aerosol concentration from two workshops 0.073 mg/m^3^ with short-term spikes to 0.908 mg/m^3^), but exposure to cobalt, sulfuric acid and chlorine are within the exposure limits.

Crane operators (group 3) work in the cabins of the overhead cranes at an elevation of 1 m during their 6-hout shifts. Up to 100% time they are exposed to nickel aerosol, but the mean concentrations of nickel, cobalt, sulfuric acid and chlorine are within OEL with some short-term peaks of nickel to 0.685 mg/m^3^. These workplaces also exhibit overexposure to noise. As opposed to the first three groups, final product cleaners (group 4) operate in a neighboring department. During their 8-h shifts, they sort final product, carry nickel plates from the conveyor, brush nickel plates, mark and pack them. These workers are overexposed to noise, exhibit much greater physical overload, lifting and carrying significant weights throughout the shift, whereas their exposure to water-soluble nickel is supplemented by the dust of non-soluble nickel (mean nickel concentrations 0.025 mg/m^3^, water non-soluble nickel 0.028 mg/m^3^ (OEL 0.05 mg/m^3^). Exposure to cobalt is below OEL, whereas chlorine and sulfuric acids are usually below the limit of detection.

Metalworkers (group 5) work 12-h shifts, and their terms of reference include, but not limited to, water, vapor, acid and chlorine pipeline maintenance, mounting new pipelines, mounting, repair and demounting acid and alkaline pumps, vacuum pump repair, candle filter cleaning, mounting and demounting crane equipment. This group is exposed to chlorine (mean 0.03 mg/m^3^), sulfuric acid (mean 0.09 mg/m^3^) and cobalt (mean 0.015 mg/m^3^); overexposed to nickel aerosol (mean concentration 0.095 mg/m^3^) and noise. Electricians (group 6) work 12-h shift, repairing electrical equipment at any location, forklifts, acid and alkaline batteries, preparing alkaline electrolyte, loading acid batteries, and final batteries assembling. Electricians are exposed to cobalt (mean concentration 0.029 mg/m^3^), chlorine (mean 0.04 mg/m^3^) and sulfuric acid (mean 0.03 mg/m^3^); and overexposed to nickel (mean 0.031 mg/m^3^ with peaks to 0.378 mg/m^3^) and noise. Lifting and carrying significant weights is also typical in this group. Finally, group 7 was compiled of other personnel, not classified at the first 6 groups due to smaller sample.

Inhalation is a major and dominant route of exposure to nickel on all stages of its production. Dermal exposure, though not as prevalent, may occur only in the electrolysis department when electrolyte drops get in contact with unprotected skin, mostly of hands.

As part of mandated routine workplace monitoring protocol, workplace air concentrations of selected pollutants, including nickel, are measured quarterly by a licensed laboratory. Methods used depend on a measured pollutant. Thus, total suspended particles and fractions are measured using gravimetric method with AФA-BП-20 filters; sulfur dioxide low concentrations, formaldehyde and arsenic anhydrates are quantitatively assessed using spectrophotometry; linear colorimetric method is usually used for carbon monoxide and higher concentrations of sulfur dioxide. Sulfuric acid, ammonia and phenol are measured with photometry, whereas polarography is used to measure nickel, cobalt and copper. In total, there are 9748 stored measurements in the database.

Industrial exposure assessment data conclude that workers of all production cycles may be exposed to higher soluble and non-soluble nickel concentrations in winter. With similar workload, air nickel concentrations in winter are higher because of no wind and air inversion typical for Far North in winter. In addition, low ambient temperatures (down to − 25–40 degrees C) necessitate slower air circulation in production buildings associated with higher costs of air conditioning, including air filtration and heating.

### Non-occupational diseases verification and occupational disease claims

At the annual screening (point 0) of this cohort observation, data on detected somatic diseases were collected and grouped into fourteen groups. Such diseases (diagnoses) were made by one or more narrow specialists, twelve of which comprised a panel of doctors to screen for fitness to work. Once identified, a specialist at screening was obliged to define whether a subject was fit to work in a given occupation as of the federal legislation. However, in the current study we needed these diagnoses to account as confounders only.

An occupational disease claim, in contrast, needed external consultation of an authorized panel of occupational doctors, which aimed to approve a case only once an association of such disease with documented exposure in the workplace was confirmed. In the Russian Federation, there exists a list of occupational diseases, which can be claimed, whereas not any suspected work-related disorder will be confirmed. Exposure data for causal association analysis is usually provided by the industrial hygiene department of an employer. A case of an occupational disease claim was a diagnosis, verified and confirmed by the governmental occupational disease council, usually on a provincial level, but sometimes referred for confirmation to Moscow. As of the legislation in the Russian Federation, a case of an occupational disease claim must have a confirmed association with the workplace exposure, and once verified is upon compensation by the employer.

In other words, to be a confirmed work-related diagnosis in the Russian Federation, an occupational disease should first sit in an approved National list of occupational diseases. Confirmation procedure starts with a “work-related disease suspicion” at the annual screening in the occupational groups with known hazards in the workplace. Such suspicion is enacted by a panel of specialists qualified in exposure assessment and occupational medicine, but not legally authorized to confirm a diagnosis or a condition to be caused by exposure at work. Diagnosis is then confirmed in a specialized medical center of occupational diseases, which concludes work-related causation of a disease. Such centers employ a range of narrow specialists and are authorized by the government to confirm a diagnosis.

### Statistical analysis

The primary outcome of interest was the cumulative incidence of occupational disease claims during twelve years of observation, both overall and in seven occupational groups. That was defined as a number of all newly diagnosed and confirmed cases of any work-related disease (diagnosis) divided by the total sample (N = 1397). In addition to the cumulative incidence, we also calculated cumulative incidence ratio for seven included groups using 2*2 tables, where unexposed groups were all remaining workers.

We screened all data for normality; and since most data were non-normally distributed, we used non-parametric tests for comparison, including Mann–Whitney U-test for continuous variables and χ2 test for binary variables in two-group comparisons. When comparing seven groups, we applied Kruskall-Wallis test. We then tested whether any of seven occupational groups were associated with a greater risk of an occupational disease claim using Cox proportional hazards regression. For that, we created seven binary variables, in which positive response was in a given occupational group, whereas the remaining were coded negative. This model was based on a time variable, defined as total years in occupation, but not just years elapsed since baseline screening in 2008. Deceased workers, those who retired and who moved were censored at a time when they left the cohort. In order to define confounders, we first tested sex, cigarette smoking (both expressed as binary variable yes/no and continuous as pack-years) and even number of detected non-occupational diseases at the annual screening. Those found associated with the outcome with p < 0.05 were included in the Cox regression model. Such modelling yielded hazard ratios (HR) with the corresponding 95% confidence interval (CI) for each of seven included occupational groups, adjusted for confounders and also based on the time elapsed since employment. All tests were considered significant with p < 0.05 and accomplished in NCSS 2021 (Utah, USA).

## Data Availability

All data generated or analysed during this study are included in this published article.
